# Supplementary material for: Detecting single gravitons with quantum sensing
Source: Nat Commun. 2024 Aug 22;15:7229. doi: 10.1038/s41467-024-51420-8 (PMC11341900; doi:10.1038/s41467-024-51420-8)
Supplement: Supplementary file 1 — Supplementary Information [file 41467_2024_51420_MOESM1_ESM.pdf]

Detecting single gravitons with quantum sensing
Supplementary Discussion

Germain Tobar,[1,2†] Sreenath K. Manikandan,[3,†] Thomas Beitel,[4] Igor Pikovski,[1,4*]

[1]Department of Physics, Stockholm University;
SE-106 91 Stockholm, Sweden.
[2] Okinawa Institute of Science and Technology;
1919-1 Tancha, Onna-son, Kunigami-gun, Okinawa, Japan 904-0495
[3] Nordita, KTH Royal Institute of Technology and Stockholm University;
SE-106 91 Stockholm, Sweden.
[4] Department of Physics, Stevens Institute of Technology;
Hoboken, NJ 07030, USA.
[†] These authors contributed equally to this work.
[*]Corresponding author, e-mail: pikovski@stevens.edu.

Gravito-phononic analogue of the photo-electric effect

S.1 Photo-electric analogy

Einstein's explanation of the photo-electric effect was a historic milestone in the development of quantum theory.[1] It showed that light deposits energy only in discrete packages, with a dependence on frequency and not intensity. Einstein realized that such behavior is not simply a property of the interaction between matter and a classical wave, but that it indicates the quantization of electromagnetic radiation itself, in apparent contradiction to Maxwell's theory.

The quantum jumps were at the time understood as intrinsic properties of atoms. The modern view is that quantum jumps are induced by the measurement apparatus as the system en-

tangles with a probe and unitary evolution is effectively transformed into an outcome.[2] The critical aspect of the photo-electric effect is that energy is discretized and is exchanged in discrete values. For our purposes, we rely on the capability to directly discern individual transitions between quantized energy levels. The reasoning is that measurement of an increase by $\hbar\omega$ in energy on the matter-sector requires by energy conservation a discrete change of energy in the same amount from the external system. In the simplest case, close to resonance and in the rotating-wave approximation, this corresponds to a $\hbar\omega$ downwards transition in energy for the gravitational field. This is the relevant regime for the cases we consider in this work, even for chirping transient sources as most of the interaction takes place close to the resonance (as computed in the methods section 'Parameter estimate' in the main text). Therefore, the gravito-phononic scheme we present is akin to the photo-electric case, in the sense that it corresponds to measurement of discrete changes in energy in the matter system from the interaction with the external wave. Rather than measuring the photo-electric current, we instead rely on the capability to determine whether a transition to the excited state has occurred.

For a monochromatic wave, and under the rotating-wave-approximation, the probability of excitation on the quantum matter becomes

$$P_{0\rightleftharpoons 1} \approx \frac{L^2}{4\pi^4}\frac{M}{\omega\hbar}h_0^2\nu^4 t^2 \mathrm{sinc}^2\left(\frac{\omega-\nu}{2}t\right), \tag{S.1}$$

and we see that in the regime of long times, the probability only becomes non-negligible once the gravitational wave frequency ν becomes close to the mechanical oscillator's frequency ω. This produces the archetypal threshold frequency - gravitational waves of frequencies lower than ω do not eject any gravito-phonons in the bar resonator, the production of gravito-phonons only occurs when the frequency of the gravitational wave is equal to that of the mechanical oscillators.

The other key feature of the photo-electric effect is the linear scaling of the absorbed energy

with incident frequency. This was famously used by Milikan to determine the value of $\hbar$ in 1916.[3] For the single mode and single transition we have considered so far, such a feature is not visible. However, one can alter the setup to include either more modes with different transition frequencies, or use more resonators at different fundamental frequencies. Monitoring their excitation probabilities in correlation with independent LIGO detections can verify the excitation from a given GW frequency, and thus show the exact same linear (but discrete) scaling with incident frequency when above threshold.

While there is a close analogy, there are also important differences between our proposed gravito-phononic scheme and the photo-electric effect. Firstly, a typical photo-electric setup involves monitoring transitions of an electron bound in a conductor, to a continuum of excited states (the free-electron with varying kinetic energies), such a continuum does not exist in our gravito-phononic detector. Next, for a photo-electric detector, the number of electrons is conserved, whereas for the gravito-phononic detector, the number of phonons is not conserved. Nevertheless at least in the regime for which the rotating-wave-approximation applies, the joint number of gravitons and phonons is conserved. This allows for the interpretation of particle conservation when energy is exchanged. In this way, despite the differences, both the photo-electric and the gravito-phononic case can be used to draw analogous conslusions in similiar regimes, namely that a discrete transition between the ground and an excited state on the matter-sector corresponds to a discrete transition on the field sector - the absorption of a $\hbar\omega$ packet of energy from the field.

S.2 Semi-classical models and energy exchange

It is crucial for our proposal that the energy eigenstates of the mechanical oscillator are continuously monitored and individually resolved, using a continuous measurement scheme such as what we propose in this article. The reason is that measuring *average* energy transfer between

the gravitational wave and the mechanical resonator is insufficient to infer discrete exchanges of energy, and thus gravitons. This is because energy transfer between a gravitational wave and the average energy of the mechanical resonator can be modelled continuously and deterministically, even under the assumption of energy conservation, as the ensemble average energy of the classical gravitational field and quantum-matter is a conserved quantity. However, the observation of discrete, individual transitions between energy eigenstates of the mechanical resonator shows the quantum nature of the process, and corresponds to the absorption of discrete energy – single gravitons from the field.

While we have argued that witnessing discrete transitions of energy on the matter sector is evidence of the absorption of a single graviton, witnessing quantum jumps in the energy level of the resonator in the strong measurement regime can also simulate the production of a photocurrent from a photoelectric sensor. The jump in the measured phonon occupation number to a set integer value (which corresponds to the production of a *gravito-phonon*, rather than a photo-electron), simulates the jump in the measured photo-current in a photo-electric detector. An analogy can be made to the archetypal feature of the photo-electric effect: the threshold frequency for a quantum jump and independence from intensity. As follows from computations in the section 'Parameter estimate' in the methods section of the main text, the transitions in matter are induced only close to the transition frequency, thus lower frequency GWs have no effect. If the GW frequency is close to resonance, the changes in energy then proceed in discrete steps whose size is independent of GW amplitude. Analogously to the photo-electric case, these signatures hint at the quantization of the field.[1]

There are, however, important loopholes in the ability to infer single field quanta, again in direct analogy to the photo-electric effect.[4,5] This is because for sufficiently large timescales, the energy input from a classical gravitational wave is greater than the $\hbar\omega_0$ transition between the ground and excited state of the mechanical resonator. Meaning that the classical gravitational

wave has more than enough energy to transfer to the mechanical resonator to account for the $\hbar\omega_0$ transition. However, such models are either non-energy conserving (the field-amplitude is unchanged after the interaction), or if the classical model includes back-action of the quantum-matter on the field, the resulting neoclassical dynamics will introduce a non-linearity due to the dependence of the classical field intensity on the quantum state of matter.[6] Our proposed experiment cannot rule out such semi-classical models, and analogously to the photo-electric case, would be open loopholes to the existence of photons. Further evidence of the quantum nature of the field can be found with the so-called time-delay argument,[7] formulated much later than the original photo-electric observations. The argument is that a classical energy transfer is continuous and takes time to build up to the observed discrete value, thus by witnessing a transition at sufficiently short times, the energy input from a classical gravitational field of intensity $I_{\rm cl}$ is insufficient to account for the discrete energy jump ($\hbar\omega_0$) on the quantum matter. However, to satisfy this condition in the gravito-phononic case, the quantum jump would need to be resolved to have occurred on a sufficiently short timescale such that the build up of energy from the classical gravitational field of intensity $I_{\rm cl}$ is smaller than $\hbar\omega_0$. For the detector that we consider in this work, such a timescale is vanishingly small, but remarkably, above the Planck scale. For example, for a $\omega_0/2\pi = 100$ Hz detector transition, with the same parameters given in the main text, the timescale for the classical energy-input to be lower than $\hbar\omega_0$ is $\tau \sim 10^{-26}$ s. This is found by solving the time for which the energy-current per unit area of the gravitational wave $j \sim \frac{c}{4}E$, for $E = \left(\frac{c^2}{32\pi G}\right)\omega_0^2 h_0^2$, accumulated over the cross-sectional-area of the cylindrical bar resonator considered in the main text, is smaller than $\hbar\omega_0$. As one can see, the time-delay argument requires the access of timescales for resolving quantum jumps which is unfeasible.

Irrespective of the model or theory for the composition of the gravitational wave – the experiment would directly show the addition and extraction of energy in discrete packets. Any

theory describing the GWs thus would need to include and account for such graviton exchange. The analogous statement holds for the electromagnetic case, and the argument involving energy conservation in combination with a semi-classical dynamics as we use here is key to some important results in modern quantum optics research. For example, to extract work from quantum measurement, it is sufficient to use semi-classical dynamics but with the crucial incorporation of the exchange of single energy quanta.[8] Even when considering large enough drive amplitudes such that these are effectively classical and the interaction Hamiltonian remains effectively unchanged, the single energy quanta have to be taken into account on both matter and field for the proper quantum thermodynamic description. Similarly, the combination of semi-classical dynamics and measurement can reveal evidence of quantumness of light[9] and can be used to infer the quantum nature of macroscopic systems.[10]

We conclude that our proposed experiment cannot serve as a proof of quantization of the field to the same extent as modern quantum optics experiments, as highlighted in the main text. For such an unambiguous confirmation of gravitons, more intricate measurements would be necessary such as confirmation of Wigner function negativity, sub-Poissonian statistics or anti-bunching. But just as it was for the electromagnetic case in 1905, due to the mutual consistency between matter and field energy transfer, our proposed setup would provide a first indication of the quantum nature of the gravitational field. While not a proof of quantization at the high threshold of modern quantum optics experiments, any description of the field will have to account for such discrete exchange of energy – if observed. Given the exceptional difficulty in observing quantum effects of gravity, showing the exchange of energy in discrete values between GWs and matter would be of similar relevance as the early photo-electric experiments for the conclusion about the quantum nature of light.

References

[1] Einstein, A. Über einem die erzeugung und verwandlung des lichtes betreffenden heuristischen gesichtspunkt. Annalen der Physik **4** (1905).

[2] Wiseman, H. M. & Gambetta, J. M. Are dynamical quantum jumps detector dependent? Physical Review Letters **108**, 220402 (2012).

[3] Millikan, R. A. A direct photoelectric determination of planck's" h". Physical Review **7**, 355 (1916).

[4] Lamb Jr, W. E. & Scully, M. O. The photoelectric effect without photons. In Polarization, Matter, Radiation, 363–369 (Presses Universitaires, Paris, 1968).

[5] Schrödinger, E. Are there quantum jumps? part i. The British Journal for the Philosophy of science **3**, 109–123 (1952).

[6] Jaynes, E. & Cummings, F. Comparison of quantum and semiclassical radiation theories with application to the beam maser. Proceedings of the IEEE **51**, 89–109 (1963).

[7] Scully, M. O. & Sargent, M. The concept of the photon. Physics Today **25N3**, 38–47 (1972).

[8] Elouard, C., Herrera-Martí, D., Huard, B. & Auffeves, A. Extracting work from quantum measurement in maxwells demon engines. Physical Review Letters **118**, 260603 (2017).

[9] Zavatta, A., Viciani, S. & Bellini, M. Quantum-to-classical transition with single-photon-added coherent states of light. Science **306**, 660–662 (2004).

[10] OConnell, A. D. et al. Quantum ground state and single-phonon control of a mechanical resonator. Nature **464**, 697–703 (2010).
